# Dislocations of the Humerus at the Shoulder

**Published:** 1895-10

**Authors:** H. B. Hill


### TEXAS MEDICAL JOURNAL.

ESTABLISHED JULY, 1885.

PUBLISHED MONTHLY.—SUBSCRIPTION $2.00 A YEAR.

VOL. XI. AUSTIN, OCTOBER, 1895. NO. 4.

### Original Contributions.

For the Texas Medical Journal.

### DISLOCATIONS OF THE HUMERUS AT THE SHOULDER,

#### With Report of Case of Four Months' Standing.

BY H. B. HILL, M. D.

(Read at Austin District Medical Society.)

MY OBJECT in writing this paper is not so much to discuss the first clause of the title, as the latter part thereof, and to place on record the case referred to. The humerus is subject to dislocation in three directions—at the shoulder—and occur in relative frequency in the order named, viz.: Forward, downward and backward. The first mentioned is divided into two sub-varieties, subcoracoid and subclavicular, and the backward dislocation into the subacromial and the subspinous; the downward variety being known as the subglenoid, which is nearly as frequent as the forward variety. It would be tedious and profitless to enter into a discussion of the symptoms and diagnosis of all the forms of this dislocation, as they are described and illustrated in the surgical works far more fully than the limits of this paper will permit. Owing to the shallowness of the glenoid cavity, the shoulder-joint is more frequently dislocated than any other, and in recent cases is usually easily diagnosed and reduced, but reduction becomes more difficult in proportion to the time that has elapsed since the occurrence of the dislocation.

The diagnosis is also difficult if swelling and inflammation have supervened, but again becomes easy after their subsidence. Occasionally it is impossible to reduce some recent cases, and some cases are difficult to keep in position after reduction has been accomplished.

The immediate effects of a dislocation consist of a rupture, more or less extensive, of the capsular ligament, with or without laceration of the other ligaments of the joint, and of neighboring tendous, muscles, vessels and nerves; in partial dislocations the capsular ligament may be merely stretched without being torn. If there is early reduction, the parts are usually gradually restored, but there may remain weakness or paralysis, with imperfect function of the limb, and a decided tendency to recurrence. If the dislocation is not reduced, the articular surfaces undergo changes. The old joint-cavity becomes filled up, its margins absorbed and flattened, while a new socket is commonly formed around the head of the dislocated bone (if it was a ball and socket-joint), which changes its shape and becomes gradually accommodated to its new position; if, however, the head of the bone rests upon muscle instead of a new socket being formed, the soft tissues undergo condensation, forming a cup-shaped cavity of fibrous structure, which becomes attached by its margins to the displaced bone and is lubricated by a synovia-like fluid. The surrounding muscles become more or less atrophied, and abnormal adhesions often form between the displaced bone and the adjacent nerves and blood vessels, a fact that should always be borne in mind in attempting reduction of old dislocations, as paralysis or hemorrhage may result from laceration thereof.

The indications of treatment in all recent cases of dislocation is to effect immediate reduction if possible; or failing to accomplish that, to attempt to convert the dislocation into the variety that offers the greatest utility of the limb in an abnormal position. The methods of reduction will be detailed in describing the procedure put into operation in attempting reduction of the ancient case that gave rise to this paper.

The question of what course to pursue in old dislocations, will depend upon the condition of each case. If a fairly useful false joint has been established, and there is not too much discomfort in the new position, it is advisable to let it alone. But it frequently happens that, owing to the extreme extent of the dislocation, that there is only the most limited motion and the greatest discomfort, on account of the tension on the muscles and ligaments, and pressure on the vessels and nerves. In such cases we are justified in making cautious attempts at reduction, and failing in that, endeavoring to convert the dislocation into one not so objectionable.

I will now give a history of the case referred to in the title of this paper:

Chas. Sinclair, white, aged about twenty years, presented himself at my office December 30, 1893, stating he had a dislocation of the shoulder-joint, which occurred about four months previously, and that two physicians had made attempts to reduce it: first on the day that it occurred, by one of them, and again the next day by both, with the use of chloroform. That the physicians said that they had succeeded in effecting reduction, but that he thought they were mistaken, as he had had several dislocations previously and knew from the condition he was in when he recovered from the effects of the chloroform, that reduction had not been accomplished. I examined the arm and found a subclavicular dislocation of the head of the humerus, the most extreme variety of forward displacement, with firm adhesions and very little motion of the arm. There was almost constant pain in the subscapular region, due to muscular tension. I told him the chances of reduction were very slight, but on account of the greatly diminished usefulness of the arm, that I would make the attempt. With the assistance of Dr. H. B. Granberry, of this city, he was chloroformed, and I proceeded as follows: First, the arm was moved and rotated in various directions, to stretch and break up the abnormal adhesions that had taken place. Then extension and counter-extension was made by placing the foot in axilla and making traction on the arm first in an outward, and then continuing the traction, while the arm was carried downward and across the chest, using the foot as a fulcrum to pry the head of the humerus into position. After several repetitions of these procedures, with various modifications, the method of manipulation was tried, following Power's Modification of Kocker's plan which is as follows: "The patient was on the back, the shoulder on the sound side held firmly down by an assistant, the injured arm grasped at the wrist and elbow, which was flexed, and the humerus brought firmly against the side, the forearm used as a lever to make outward rotation until it pointed directly outward, the elbow was then carried along the front of the chest to the median line, when with a quick inward rotation the hand was carried to opposite shoulder." The only result of these maneuvers was to convert the subclavicular into a subcoracoid dislocation, and a partial breaking up of the adhesions. We then passed a folded cloth beneath the axilla with which counter-extension was made by traction on the arm directly outward, and followed by downward traction with the foot in the axilla, when we were gratified to perceive the arm return to its natural position with a decided snap. To say that it had returned to its natural position is only partially correct; for the glenoid cavity having become filled up, the head of the bone rested on a flat surface, instead of the cup shaped depression it normally occupies. Consequently when Dr. Granberry made an upward movement of the arm to test the accuracy of the reduction, the dislocation immediately recurred, and was again reduced by the procedure last described. A careful examination showed that the head of the bone rested too far forward, to remedy which the following procedure was resorted to: A long folded towel was passed around the arm at the axilla, and traction was made with this obliquely outward and backward, while counter-traction was made with the band under the axilla, while at the same time I made pressure in long axis of the humerus upon the elbow with one hand, and with the other guided the head of the bone into its proper position. We were as successful as was possible, considering the changes that had occurred, owing to the long standing of dislocation. Among other changes, a great degree of atrophy had occurred in the deltoid muscle, which I thought to be due to want of use, but which may have been due to nerve injury, causing paralysis. We placed on a bandage designed to prevent a recurrence of the dislocation, and on seeing him the next day, there was very little swelling or inflammation, and with the exception of a persistent chloroform nausea, he expressed himself as feeling much more comfortable than previous to his reduction. He left for his home, which was about thirty miles distant, promising to return in five or six days, or earlier, if there was any recurrence of the dislocation, but failed to do so until sixteen days had elapsed; when he did so we found the bandage displaced, loose and stretched; that dislocation had recurred, and apparently as firmly bound down as when first seen. To be as brief as possible, we had much difficulty in accomplishing the second reduction, but finally succeeded by similar measures to those resorted to at first. After which we applied a bandage so arranged that recurrence was absolutely impossible so long as it was retained in place, and to prevent any displacement, I stitched it at numerous points. I required him to remain in the city for one week longer, when he again returned home, and so far has had no return of the displacement. When last heard from there was a very decided increase of movement in the arm, but still quite marked atrophy and loss of power in the deltiod, which I am endeavoring to remedy, by massage and passive as well as voluntary motion.

There are various other methods of reducing dislocations of the shoulder joint, which will only be briefly referred to. White, Mothe, and others made extension by drawing the arm upward, counter-extension being made by the foot or hand placed above the acromion process.

By leverage: The patient being seated in a chair, the foot of the operator rests on the edge of the chair, the knee in the axilla as a fulcrum, the arm being pressed downward as a lever, the head of the humerus is pried into position. Subacromial and subspinous dislocations may be reduced by extension and counter-extension in the direction of the displacement, counter-extension being made by the opposite arm or the band in axilla. Cases either of recent or ancient disclocation that can not be reduced by some of the various methods of treatment described, may where the usefulness of the arm is so limited as to make it advisable, be treated by incision and the reduction being thus accomplished, excising the head of the humerus if necessary. Of course such an operation would require the most careful antisepsis, and a thorough knowledge of the anatomy of the joint. The various accidents that may occur during efforts at reduction of old dislocation such as fracture, rupture of muscles, and laceration of blood-vessels, etc., require a cessation of all efforts, and treatment according to the nature of the accident.

